# Association between cervical artery dissection and spinal manipulative therapy –a medicare claims analysis

**DOI:** 10.1186/s12877-022-03495-5

**Published:** 2022-11-29

**Authors:** James M Whedon, Curtis L Petersen, Zhongze Li, William J Schoelkopf, Scott Haldeman, Todd A MacKenzie, Jon D Lurie

**Affiliations:** 1 Health Services Research, Southern California University of Health Sciences, 16200 Amber Valley Drive, 90604 Whittier, CA USA; 2 The Dartmouth Institute for Health Policy and Clinical Practice, Dartmouth College, Hanover, NH USA; 3 Northern Light Health, Portland, ME USA; 4 Department of Neurology, University of California at Irvine, Irvine, CA USA

**Keywords:** Cervical spine, Spinal manipulation, Cervical artery dissection, Vertebral artery dissection, Carotid artery dissection; ischemic stroke, Chiropractic

## Abstract

**Background:**

Cervical artery dissection and subsequent ischemic stroke is the most serious safety concern associated with cervical spinal manipulation.

**Methods:**

We evaluated the association between cervical spinal manipulation and cervical artery dissection among older Medicare beneficiaries in the United States. We employed case-control and case-crossover designs in the analysis of claims data for individuals aged 65+, continuously enrolled in Medicare Part A (covering hospitalizations) and Part B (covering outpatient encounters) for at least two consecutive years during 2007–2015. The primary exposure was cervical spinal manipulation; the secondary exposure was a clinical encounter for evaluation and management for neck pain or headache. We created a 3-level categorical variable, (1) any cervical spinal manipulation, 2) evaluation and management but no cervical spinal manipulation and (3) neither cervical spinal manipulation nor evaluation and management. The primary outcomes were occurrence of cervical artery dissection, either (1) vertebral artery dissection or (2) carotid artery dissection. The cases had a new primary diagnosis on at least one inpatient hospital claim or primary/secondary diagnosis for outpatient claims on at least two separate days. Cases were compared to 3 different control groups: (1) matched population controls having at least one claim in the same year as the case; (2) ischemic stroke controls without cervical artery dissection; and (3) case-crossover analysis comparing cases to themselves in the time period 6–7 months prior to their cervical artery dissection. We made each comparison across three different time frames: up to (1) 7 days; (2) 14 days; and (3) 30 days prior to index event.

**Results:**

The odds of cervical spinal manipulation versus evaluation and management did not significantly differ between vertebral artery dissection cases and any of the control groups at any of the timepoints (ORs 0.84 to 1.88; p > 0.05). Results for carotid artery dissection cases were similar.

**Conclusion:**

Among Medicare beneficiaries aged 65 and older who received cervical spinal manipulation, the risk of cervical artery dissection is no greater than that among control groups.

**Supplementary Information:**

The online version contains supplementary material available at 10.1186/s12877-022-03495-5.

## Background

Cervical artery dissection (CeAD) is a potentially serious condition that occurs when weakening and disruption of the arterial lining allow blood to get in between and separate the layers of the arterial wall. Patients with CeAD often present with neck pain or headache, may be asymptomatic or present as a stroke in progress. The blood that accumulates within the arterial wall can occlude the artery or cause a blood clot that in turn can be dislodged, leading to an ischemic stroke. CeAD can occur in either the carotid or vertebral arteries [1]. Although reporting of the incidence of CeAD has increased in recent years this is felt likely due to increased sensitivity and increased availability and utilization of imaging technologies [2]. CeAD remains uncommon, with an incidence reported to be approximately 3 per 100,000 for carotid artery dissection (CAD) and 1 per 100,000 for vertebral artery dissection (VAD) [3], accounting for approximately 2% of all ischemic strokes [4, 5]. In a recent case-control study of 165 patients with ischemic strokes, age < 50 years old, headache, and neck pain positively correlated with a diagnosis of CeAD, and those symptoms were a common prodrome of CeAD [6]. Blunt or penetrating trauma can precede CeAD but the etiology of spontaneous CeAD is unclear [7, 8]. A systematic review of the risk factors for CeAD evaluated 31 case control studies published between 1980 and 2005 and found strong associations between CeAD-associated stroke and ‘trivial trauma’ in the form of manipulation of the cervical spine (adjusted OR = 3.8, 95% CI = 1.3–11.0) [9]. 

Although CADs are generally more common than VADs, there is a reported 3:1 predominance of VADs vs. CADs following cervical spine manipulation (CSM) [10]. It has been hypothesized that CSM can cause VAD through rotation and extension of the neck that stretches the vessel where it penetrates either the atlas or posterior atlanto-occipital membrane [11]. However, no direct evidence has been found to support this hypothesis, and a retrospective review of 64 medical legal cases of stroke temporally associated with CSM found no apparent dose-response relationship between CSM and CeAD [12].

CSM is commonly used to treat neck pain, with an estimated 1.7 million older Medicare beneficiaries receiving chiropractic spinal manipulation services in 2008 [13]. A recent systematic review found that CSM is an effective treatment for chronic nonspecific neck pain [14], and The American College of Physicians, the American Pain Society [15], the Task Force on Neck Pain and its Associated Disorders [16], and the American Geriatric Society recommend CSM for managing neck pain in older adults. 17 However, the American Heart Association (AHA) and the American Stroke Association (ASA) have issued recommendations that patients should be informed of the association between CSM and CeAD [18]. The risk of CeAD is the most significant safety concern regarding CSM and a recent systematic review found that much larger sample sizes would be required to fully assess the safety of CSM [14]. The availability of very large datasets of Medicare health claims presents an opportunity to conduct analyses with sufficient power to quantify the risk of CAD associated with CSM.

Although generally considered a problem in younger, middle-aged patients, recent studies suggest that CeAD may be overlooked and underdiagnosed in older patients [19, 20]. A large epidemiologic study utilizing data from the National Inpatient Sample found that while CeAD accounted for a higher proportion of hospitalization for ischemic stroke in younger age groups, the actual prevalence of CeAD related stroke hospitalization increased with age. 21 As such, rigorous examination of the potential relationship between CSM and CeAD in older Medicare beneficiaries seems warranted. In this study, to better inform US policymakers, providers, and patients, we evaluated the association between CSM and CeAD among Medicare beneficiaries. Using claims data from 2007 to 2015 for fee-for-service Medicare enrollees aged 65–99, we determined the association between CSM and CeAD using several different control groups and advanced statistical approaches to control for potential confounding.

## Methods

### Design

**Fig. 1:**
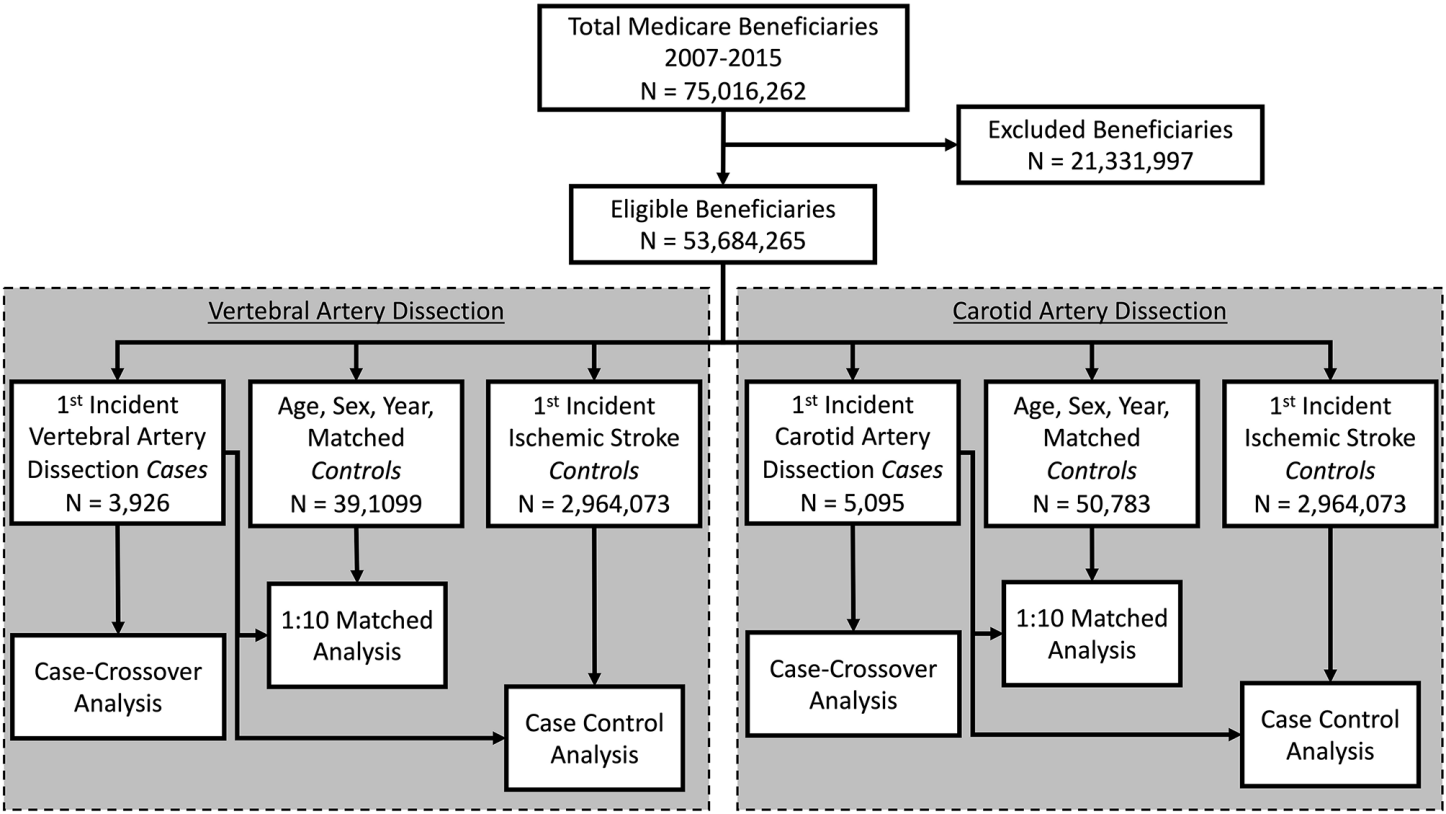
Definition of Cases and Controls. The design of the study consists of three case-controls drawn from Medicare beneficiaries between 2007 and 2015. It studies VAD and CAD cases and compares each to (i) age-sex-year matched Medicare beneficiaries (10:1), (ii) Ischemic stroke controls and self-controls (cases 6 months before their incident artery dissection). The numbers of each case and control are shown.

As depicted in Fig. 1 we analyzed the relationship between CSM and CeAD with three different types of controls: (1) a case-control design consisting of cases with CeAD and controls from the general population of Medicare beneficiaries matched by sex, age (in years) and calendar year of the CeAD; (2), a case-control design with the same cases and controls with ischemic stroke from the population of Medicare beneficiaries; and (3) a case-crossover design, in which exposures prior to the CeAD are compared to exposures in the time period 6 months earlier in the same patient.

### Population

The study subjects included fee-for-service Medicare beneficiaries using 100% 2007–2015 Medicare Part A (covering hospitalizations) and B (covering outpatient encounters and physician services) files. We included beneficiaries aged 65 and older with at least one Part B claim in a calendar year with the following annual exclusions: (a) any Medicare Advantage; (b) less than full Part B enrollment for the entire calendar year (or from the month turning 65 to month of death); and (c) residence outside the 50 United States or Washington, DC. All subjects were concurrently and continuously enrolled in Medicare Parts A and B for at least two consecutive years.

### The cases

The primary outcome was the occurrence of a CeAD which was sub-divided into (1) VAD and (2) CAD. The cases were identified as beneficiaries with a new (not recorded in the prior year) diagnosis of International Classification of Disease (ICD-9) code 443.24 (VAD) or 443.21 (CAD) in the primary diagnosis field on at least one inpatient hospital claim or primary/secondary diagnosis for outpatient hospital and Part B claims on at least two separate days. The majority (73%) of CeAD cases had a diagnosis of stroke within 30 days of the CeAD diagnosis.

### The controls

As discussed above, there were three types of controls. For population controls, we matched Medicare beneficiaries without CeAD, which we refer to as population controls, to the CeAD cases in a 10:1 ratio. Controls were matched for age (in years), sex and having at least one claim on the same day (+/- 1 week) as the case. Controls were excluded if they ever had a diagnosis of CeAD. For the ischemic stroke controls, we identified beneficiaries with a diagnosis code for non-CeAD-associated ischemic stroke (ICD9 codes 431, 432, 434, 433.10, or 433.11) in the primary diagnosis field on at least one inpatient hospital claim or primary/secondary diagnosis for outpatient hospital and Part B claims on at least two separate days. For the case-crossover study we evaluated claims for the CeAD cases in the corresponding time period 6–7 months prior to their CeAD.

### Index date

The index date is defined as the date of diagnosis of CeAD in the cases, as the date of the corresponding claim in the population controls, and as the date of diagnosis of ischemic stroke in the stroke controls. In the case-crossover analysis, the index date for the control period is the date 180 days prior to the occurrence of CeAD.

### Primary and secondary exposures

The primary exposure was CSM, as identified by Current Procedural Terminology (CPT) codes 98,940–98,942 (indicating spinal manipulation by a doctor of chiropractic) associated with a primary diagnosis of headache (ICD-9 code 339.xx) or neck pain (ICD-9 codes 721.0, 721.1, 722.0, 722.4, 722.71, 722.81, 722.91, 723.1-723.8, 739.1, 756.16, 839.0x, 847.0, 953.0, or 953.4) or other disorders of the head or neck pain that are commonly treated by spinal manipulation (ICD-9 codes 739.1, 723.1, 739.0, 722.4, 839.xx, 723.3, 847.0, or 839.00) in order to try to localize the manipulation to the cervical region. The secondary exposure was the occurrence of an encounter for Evaluation and Management (E&M) as indicated by ICD-9 codes 99,201–99,205 and 99,211–99,215 with the same associated diagnoses discussed above. Using the primary and secondary exposure we created a 3-level categorical variable, (i) CSM, (ii) E&M but no CSM and (iii) neither CSM nor E&M. The E&M only category was selected to the referent group. Individuals with both a CSM and an E&M visit in the requisite time period were analyzed in the CSM group.

### Timeframes for the exposure

We created the 3-level exposure described above for each of the following time frames, up to 7, 14 and 30 days prior to the index event. For instance, for the 7-day time window, the 3-level categorical exposure is (i) CSM in the 7 days before index, (ii) E&M but no CSM in the 7 days before index, and (iii) neither CSM nor E&M in the 7 days before index.

### Covariates

Covariates included demographics age, sex, race (categorized as White, Black, Asian, Hispanic, North American Native, and Other) and calendar year. In addition, to control for comorbidities we considered all diagnoses 14 to 365 days preceding the index date grouped using the Multi-level Clinical Classification Software (CCS) of the Healthcare Cost and Utilization Project [22]. ICD-9 codes were grouped into categories using the third level of the multi-level classification.

### Statistical methods

We used odds ratios to characterize the association between CeAD and the 3-level exposure, CSM vs. E&M vs. neither, with E&M set as the referent group. To control for the covariates described above we applied multivariable logistic regression to the dataset consisting of CeAD cases and ischemic stroke controls. To control for covariates in the analysis comparing CeAD cases to matched population controls we used multivariable conditional logistic regression. The conditional logistic regression estimates the odds ratios conditional on the sex-age-year matches, in addition to race and diagnostic covariates. Sex, age and calendar year therefore have null coefficients due to matching on them.

Due to large number of diagnostic covariates (over 430) we used variable selection methods. To select predictors of CeAD cases versus ischemic stroke controls we employed Least Absolute Shrinkage and Selection Operator (LASSO) for logistic regression to select covariates [23]. In particular, we selected those covariates that had a nonzero LASSO coefficient. The penalty parameter in the LASSO was determined using 10-fold cross-validation with optimization of the binomial deviance (analogous to log-likelihood). To select predictors of CeAD cases versus population controls that accounts for matching by sex, age, and year, we employed stepwise conditional logistic regression (we are not aware of a LASSO adaptation to conditional logistic regression). This approach to covariate selection served to identify any comorbidities that predict diagnosis of CeAD and may act as confounders.

We tested if there was an association of the exposure (CSM vs. E&M vs. neither) with CeAD in the case-crossover analysis using conditional logistic regression conditioning on subject (e.g., the pair of observations from CeAD and 6 months earlier).

All analyses above were repeated for each of the three time frames for exposure to CSM and E&M (7, 14 and 30 days). That is, we report odds ratios comparing CSM to E&M and neither CSM nor E&M to E&M exposure in each of these time periods.

As yet another perspective, we employed a propensity score approach to estimate the odds ratio relating CeAD to CSM. This consisted of the following steps. Using data from the population controls we modeled the occurrence of CSM in the 7 days before index as a function of demographics and comorbidities using logistic regression. The predicted probabilities (fitted values) from this logistic regression were used to calculate the inverse weighted propensities. The final step was to employ a weighted logistic regression to estimate the odds ratio relating VAD (or CAD) to CSM in the previous 7 days.

Our study is powered (at 80%) to detect odds ratios of VAD with CSM in the previous week (relative to E&M) for the population of 2.0. The corresponding detectable odds ratio for CAD with CSM is 1.8. The detectable odds ratios using the Ischemic stroke controls are slightly smaller as we had more than 10 of those controls per CAD case. Statistical software employed was SAS 9.4, and R (including libraries, tidyverse, & glmnet).

## Results

Figure 1 displays the three groups of patients drawn from all Medicare beneficiaries between 2007 and 2015, individuals who incurred a CeAD, those who experienced an ischemic stroke, and population controls. Total CeAD cases, population controls, and ischemic stroke controls numbered 9,021, 89,892, and 2,964,073, respectively. The number of cases used in the population-matched analyses was reduced due to not finding 10 matches for those cases.

Table 1 displays descriptive statistics for the VAD and CAD cases, as well the Ischemic stroke and population controls. There were no major demographic differences between any of the populations.

**Table 1:** Patient Characteristics

Demographics	VAD Cases (n = 3,926)	VAD-matched Population Controls (n = 39,109)	CAD Cases (n = 5,095)	CAD-matched Population Controls (n = 50,783)	Ischemic Stroke Controls (n = 2,964,073)
**Age (mean, SD)**	71.17 (12.87)	71.16 (12.87)	70.18 (12.80)	70.18 (12.8)	75.37 (11.22)
**Race (n, %)**					
White	3,397 (86.79)	32,831 (83.95)	4,279 (84.32)	42,804 (84.29)	2,415,527 (81.62)
Black	294 (7.51)	3,743 (9.57)	527 (10.38)	4878 (9.61)	390,799 (13.21)
Asian	72 (1.84)	718 (1.84)	86 (1.69)	905 (1.78)	46,684 (1.58)
Hispanic	59 (1.51)	854 (2.18)	84 (1.66)	1,005 (1.98)	58,771 (1.99)
Native American	20 (0.51)	189 (0.48)	20 (0.39)	254 (0.50)	13,031 (0.44)
Other	72 (1.84)	535 (1.37)	79 (1.56)	676 (1.33)	34,562 (1.17)
**Gender (n, %)**					
Male	2,067 (52.65)	20,571 (52.6)	2,628 (51.58)	26,209 (51.61)	1,308,192 (44.08)
Female	1,859 (47.35)	18,538 (47.4)	2,467 (48.42)	24,574 (48.39)	1,659,762 (55.92)

VAD: Vertebral Artery Dissection; CAD: Carotid Artery Dissection; SD: Standard Deviation

Table 2 characterizes the association of VAD and CAD with CSM in the 1 week, 2 weeks, and 30 days prior to the index date compared to the Medicare population controls. The odds of CSM compared to E&M did not significantly differ between VAD cases and controls at any of the time points. The odds of CSM compared to E&M were 1.60 (95% CI: 0.66, 3.89) higher in VAD patients within the previous 7 days but this finding is not statistically significant (P > 0.10). The odds of having neither CSM nor E&M in the prior 14- and 30-day windows were significantly lower among VAD cases, likely representing care seeking behavior for symptoms. Similarly, the odds of CSM relative to E&M were not significantly elevated in CAD cases compared to controls in either the previous 7, 14 or 30 days; no significant differences between CAD cases and controls were found for having neither CSM nor E&M at any of the time points either. Odds ratios for race, diagnostic covariates, and all comorbidities in the population-matched analyses are displayed in supplementary **Table S1**.

**Table 2:** Population Controls

	Vertebral Artery Dissection				Carotid Artery Dissection			
**Exposure**	**n cases**	**n controls**	**Odds Ratios (95% CI)**	**P-value**	**n cases**	**n controls**	**Odds Ratios (95% CI)**	**P-value**
**Exposure - Past 7 days**								
E&M	92	349	**1**		82	497	**1**	
CSM	45	161	1.60 (0.66, 3.89)	0.30	20	82	0.75 (0.31, 1.82)	0.53
Neither CSM nor E&M	3,789	38,599	0.75 (0.46, 1.23)	0.25	4,993	50,081	1.26 (0.85, 1.86)	0.26
**Exposure - Past 14 days**								
E&M	142	455	**1**		146	652	**1**	
CSM	66	251	1.47 (0.70, 3.10)	0.31	34	299	1.15 (0.58, 2.30)	0.68
Neither CSM nor E&M	3,718	38,403	0.64 (0.42, 0.99)	0.042	4,915	49,832	1 (0.73, 1.37)	0.98
**Exposure - Past 30 days**								
E&M	202	700	**1**		215	971	**1**	
CSM	83	373	1.43 (0.76, 2.71)	0.27	68	446	1.74 (1.03, 2.94)	0.040
Neither CSM nor E&M	3,641	38,036	0.69 (0.49, 0.97)	0.034	4,812	49,366	0.95 (0.73, 1.25)	0.72

*E&M: Evaluation and Management for complaint of neck pain; CSM: Cervical Spinal Manipulation;*

*n = number of subjects; CI = confidence interval*

The inverse weighted propensity estimate of the odds ratio relating VAD to CSM in the previous 7 days (relative to E&M only) was 0.73 (95%CI: 0.43–1.24). The corresponding inverse weighted propensity odds ratio for CAD was 0.79 (95%CI; 0.29, 2.11).

Table 3 examines the association of CSM with VAD and CAD compared to the ischemic stroke controls. Compared to those with an E&M visit only, the odds of CSM in VAD cases versus non-CeAD ischemic stroke controls were not significantly different at any of the time points (e.g., 7 days - OR 0.84; 95% CI 0.60, 1.17). The odds of having neither a CSM nor E&M visit were markedly lower in the VAD group at every time point (ORs 0.36 to 0.39; p < 0.001). The odds of CSM versus E&M were significantly lower at each time point for CAD cases versus non-CeAD ischemic stroke controls (e.g., 7 days - OR 0.68; 95% CI 0.45, 0.98). The ORs for exposure within 14 and 30 days were similar. As with VAD, the odds of receiving neither CSM nor E&M among CAD cases versus ischemic stroke controls was significantly lower (P < 0.0001) than the reference exposure (E&M) in all three time intervals. The ORs for sex, age, race, year and diagnostic covariates for the ischemic stroke control analysis are displayed in supplementary **Table S2**.

**Table 3:** Ischemic Stroke Controls

Exposure Exposure	n controls	Vertebral Artery Dissection			Carotid Artery Dissection		
		**n Cases**	**Odds Ratios (95% Confidence Interval)**	**P-value**	**n Cases**	**Odds Ratios (95% Confidence Interval)**	**P-value**
**Exposure Past 7 days**							
E&M	16,052	94	1.00		88	1.00	
CSM	8,223	51	0.84 (0.60, 1.17)	0.31	30	0.68 (0.45, 0.98)	0.047
Neither CSM nor E&M idays	2,945,156	4,411	0.36 (0.30, 0.44)	< 0.0001	5,824	0.52 (0.43, 0.64)	< 0.0001
**Exposure Past 14 days**							
E&M	27,167	150	1.00		150	1.00	
CSM	12,691	73	0.90 (0.68, 1.18)	0.46	46	0.70 (0.52, 0.94)	0.02
Neither CSM nor E&M days	2,929,573	4,333	0.39 (0.34, 0.46)	< 0.0001	5,746	0.49 (0.42, 0.56)	< 0.0001
**Exposure Past 30 days**							
E&M	46,416	205	1.00		216	1.00	
CSM	19,225	89	0.84 (0.60, 1.17)	0.31	79	0.68 (0.45, 0.98)	0.05
Neither CSM nor E&M days	2,903,790	4,262	0.36 (0.30, 0.44)	< 0.0001	5,647	0.52 (0.43, 0.64)	< 0.0001

E&M: Evaluation and Management for complaint of neck pain; CSM: Cervical Spinal Manipulation

Table 4 shows the results of the case-crossover analyses. CSM was not more likely than E&M in the 7 days prior to a VAD (P = 0.31), nor in the prior 14 days (P = 0.75) or prior 30 days (P = 0.11). For CAD, there was no increased risk of receiving CSM compared to E&M in any of the time periods; in fact, point estimates suggest possible reduced risk (ORs 0.38 to 0.88) although this was only statistically significant for the 14-day time point.

**Table 4:** Case Crossover Analysis

	Vertebral Artery Dissection			Carotid Artery Dissection		
**Exposure**	**n**	**Odds Ratios (95% Confidence Interval)**	**P-value**	**n**	**Odds Ratios (95% Confidence Interval)**	**P-value**
**Exposure Past 7 days**						
E&M	94	1.00		88	1.00	
Neither CSM nor E&M in 7 days	51	0.20 (0.12–0.33)	< 0.001	30	0.31 (0.20–0.49)	< 0.001
CSM in prior 7 days	4,411	1.59 (0.65–3.85)	0.31	5,824	0.48 (0.22–1.04)	0.06
**Exposure past 14 days**						
E&M past 14 days	150	1.00		150	1.00	
Neither CSM nor E&M in 14 days	73	0.20 (0.14–0.31)	< 0.001	46	0.30 (0.21–0.43)	< 0.001
CSM in prior 14 days	4,333	0.90 (0.46–1.74)	0.75	5,746	0.38 (0.20–0.70)	0.002
**Exposure past 30 days**						
E&M past 30 days	205	1.00		216	1.00	
Neither CSM nor E&M in 30 days	89	0.22 (0.16–0.31)	< 0.001	79	0.41 (0.31–0.53)	< 0.001
CSM only in 30 days	4,262	0.63 (0.36–1.11)	0.11	5,647	0.88 (0.51–1.50)	0.63

E&M: Evaluation and Management for complaint of neck pain; CSM: Cervical Spinal Manipulation

**Fig. 2:**
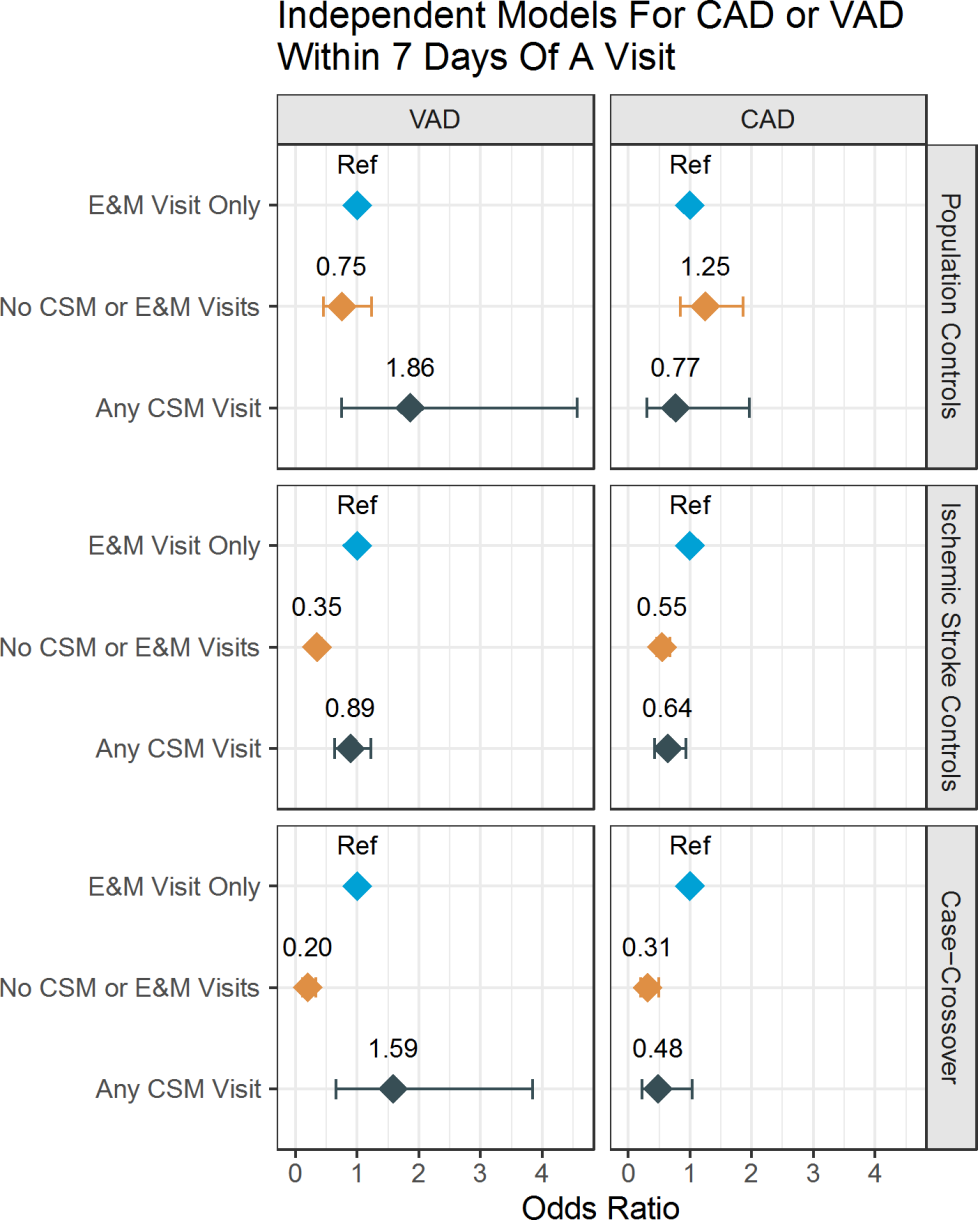
Association Of CeAD And encounter type by analytic approach. The Forest Plot displays odds ratios for the association of VAD and CAD with receiving - in the 7 days prior to diagnosis of CeAD - CSM, or neither CSM nor E&M, vs. E&M (referent group). The odds ratios are reported for three types of design: Medicare population case-controls, ischemic stroke controls, and case-crossover (the control is a 6 month look back in the case).

Figure 2 visualizes the odds ratios across all three types of study designs (i) population controls, (ii) Ischemic Stroke controls and (iii) case-crossover controls (6-month look back) relating the odds of VAD and CAD to exposure in the previous 7 days of either CSM, E&M (referent) and neither.

## Discussion

Prior to this research project, the largest study of the association of CSM with CeAD consisted of 966 cases [24]. In this study, the sample size was more than 9 times as large at 9,021, providing a statistical advantage for studying an uncommon condition [25]. This was also the first study to examine the relationship between CSM and CAD specifically in older adults, who tend to be co- morbid, at risk of stroke in general, and at risk for the adverse effects of analgesic medications (the primary alternative to CSM for neck pain). The various analyses consistently fail to show any increased risk associated with CSM and a fairly consistent pattern of reduced risk associated with neither CSM nor E&M. As a possible explanation for the association between CSM and VAD, it has been hypothesized that the onset of neck pain may represent a dissection in progress, which causes a patient to seek CSM [12]. Our findings are consistent with this hypothesis, with patients seeking out treatment for neck related symptoms leading up to their diagnosis of CeAD, as evidenced by the consistently low OR for the group with neither CSM nor E&M.

Only four studies [24, 26–28] were large enough to inform the AHA/ASA position paper on the association between CSM and CeAD [18]. These studies have been criticized for controls which, although age- and sex-matched, were much healthier than the cases [29]. Further, the two largest studies were based on the same dataset from the 1990s, which may have misidentified CeAD [30]. While these 2 studies found a strong relationship between CSM and posterior circulation stroke among patients aged 45 or younger, a temporal association of CSM preceding stroke was not demonstrated; moreover, a lack of adjustment for comorbidities may have obscured a similar relationship in older patients. In the present study, we used advanced statistical techniques to control for confounders, including comorbidities, used multiple control groups, and compared CSM to E&M to control for care-seeking behavior leading up to a diagnosis of CeAD and found no significant relationship between CSM and CeAD in this population, but did find a strong and consistent relationship between being seen for head or neck complaints (either CSM or E&M versus neither) and a subsequent diagnosis of CeAD.

A previous study that found no significant relationship between CSM and any stroke used only a single year of Medicare claims data (2008) and was not adequately powered to examine specifically CeAD-associated stroke, which is the only type of stroke hypothesized to be related to CSM [31]. In the present study, we leveraged the statistical power of analyzing a multi-year Medicare claims dataset and found no significant relationship between CSM and either VAD or CAD.

Our study is characterized by advanced statistical methods to control for potential confounding, three different control groups (ischemic stroke controls, population controls and a case-crossover analysis), multiple different time points for the exposure (7, 14 and 30 days), as well as controlling for care seeking behavior by comparing the risk of CeAD in patients receiving CSM to patients receiving a similar E&M visit or neither. Our finding strongly suggest that CeAD patients are likely seeking out care for neck pain and related symptoms from either a CSM provider, medical provider or both in the period leading up to their diagnosis of CeAD rather than having a specific risk for CeAD imparted by receipt of CSM in this population.

### Limitations

General limitations of using health claims data for research include inconsistencies in billing practices and coding of procedures. Because there is no procedure code specific to CSM, we identified CSM as spinal manipulation in patients with neck pain and related diagnoses. Because this was a retrospective study, the subjects were not randomized and there may have been systematic difference between the groups. However, we attempted to minimize any confounding through use of advanced statistical methods to control for differences between the groups, the use of multiple different control groups (including the case-crossover analysis which uses each case as their own control), and the use of multiple different time points. Despite the high statistical power, the large confidence interval in Fig. 2 for “Any CSM Visit” may suggest uncertainty about the results. Finally, we note that this study was limited to individuals aged 65 and older; subsequent research should investigate the association between CSM and CeAD among adults under age 65.

## Conclusion

Among Medicare beneficiaries aged 65 and older who received cervical spine manipulation, the association with cervical artery dissection is no greater than that among the control groups, and CSM does not appear to be a significant risk factor for CeAD in this population group.

## Electronic supplementary material

Below is the link to the electronic supplementary material.

Supplementary Material 1: Table S1. Medicare Population Odds Ratios Relating Cervical Artery Dissection to Age, Sex, Race, and Diagnostic Covariates used in the Multi-variable Adjustment. Table S2. Ischemic Stroke Controls Odds Ratios for Variables used in the Multi-variable Adjustment.

## Data Availability

The data that support the findings of this study were obtained from The Centers for Medicare and Medicaid Services (CMS), United States. Department of Health and Human Services. These data were accessed by the principal investigator under the terms of a data use agreement with CMS, which strictly prohibits sharing of data; thus, the CMS datasets are not publicly available. To request data from this study please contact Dr. Todd Mackenzie: Todd.A.MacKenzie @dartmouth.edu.

## Declarations

All methods were carried out in accordance with relevant guidelines and regulations.

## Abbreviations

AHA: American Heart Association.
ASA: American Stroke Association.
CAD: Carotid Artery Dissection.
CCS: Clinical Classifications Software.
CeAD: Cervical Artery Dissection.
CPT: Current Procedural Terminology.
CSM: Cervical Spinal Manipulation.
E&M: Evaluation and Management.
ICD-9: International Classification of Diseases, Ninth Revision.
LASSO: Least Absolute Shrinkage and Selection Operator.
OR: Odds ratio.
P: Probability.
VAD: Vertebral Artery Dissection.
