# Supplementary material for: Association between cervical artery dissection and spinal manipulative therapy –a medicare claims analysis
Source: BMC Geriatr. 2022 Nov 29;22:917. doi: 10.1186/s12877-022-03495-5 (PMC9710172; doi:10.1186/s12877-022-03495-5)
Supplement: Supplementary file 1 — Supplementary Material 1: Table S1. Medicare Population Odds Ratios Relating Cervical Artery Dissection to Age, Sex, Race, and Diagnostic Covariates used in the Multi-variable Adjustment. Table S2. Ischemic Stroke Controls Odds Ratios for Variables used in the Multi-variable Adjustment. [file 12877_2022_3495_MOESM1_ESM.docx]

**Table S1. Medicare Population Odds Ratios Relating Cervical Artery Dissection to Age, Sex, Race, and Diagnostic Covariates used in the Multi-variable Adjustment**

| **Variables** | **Odds Ratios (95% Confidence Interval)** | **P-value** | **Variables** | **Odds Ratios (95% Confidence Interval)** | **P-value** |
| --- | --- | --- | --- | --- | --- |
| Age | 1 (NA) | NA | Age | 1 (NA) | NA |
| Male | 1 (NA) | NA | Male | 1 (NA) | NA |
| Race (White set as a reference) |  |  | Race (White set as a reference) |  |  |
| Unknown | 0.96 (0.37, 2.45) | 0.927 | Unknown | 0.76 (0.37, 1.56) | 0.455 |
| Black | 0.80 (0.61, 1.04) | 0.091 | Black | 1.24 (1.05, 1.46) | 0.009 |
| Other | 1.45 (0.8, 2.62) | 0.218 | Other | 1.22 (0.81, 1.82) | 0.341 |
| Asian | 0.80 (0.46, 1.39) | 0.418 | Asian | 1.14 (0.79, 1.64) | 0.493 |
| Hispanic | 0.88 (0.51, 1.52) | 0.65 | Hispanic | 0.83 (0.56, 1.22) | 0.341 |
| North American Native | 0.42 (0.13, 1.38) | 0.152 | North American Native | 0.70 (0.32, 1.53) | 0.372 |
| Year | 1 (NA) | NA | Year | 1 (NA) | NA |
| Diagnostics |  |  | Diagnostics |  |  |
| Acute and chronic tonsillitis [124.] | 5.40 (1.62, 17.96) | 0.006 | Acute cerebrovascular disease [109.] | 7.46 (6.43, 8.64) | <0.0001 |
| Acute cerebrovascular disease [109.] | 23.03 (18.74, 28.3) | <0.0001 | Acute posthemorrhagic anemia [60.] | 1.60 (1.22, 2.09) | <0.001 |
| Acute myocardial infarction [100.] | 2.21 (1.49, 3.28) | <0.0001 | All other congenital anomalies | 5.68 (3.46, 9.33) | <0.0001 |
| All other congenital anomalies | 2.50 (1.11, 5.61) | 0.026 | Allergic reactions [253.] | 1.45 (1.26, 1.66) | <0.0001 |
| Aortic and peripheral arterial embolism or thrombosis [116.] | 3.09 (1.69, 5.65) | <0.001 | Aortic and peripheral arterial embolism or thrombosis [116.] | 2.56 (1.81, 3.61) | <0.0001 |
| Aortic; peripheral; and visceral artery aneurysms [115.] | 3.33 (2.47, 4.48) | <0.0001 | Aortic; peripheral; and visceral artery aneurysms [115.] | 6.22 (5.28, 7.33) | <0.0001 |
| Aspiration pneumonitis; food/vomitus [129.] | 2.34 (1.41, 3.88) | 0.001 | Blindness and vision defects [89.] | 1.60 (1.38, 1.85) | <0.0001 |
| Blindness and vision defects [89.] | 1.56 (1.27, 1.93) | <0.0001 | Cancer of bladder [32.] | 0.59 (0.36, 0.95) | 0.029 |
| Calculus of urinary tract [160.] | 0.49 (0.33, 0.73) | <0.001 | Cancer of breast [24.] | 0.66 (0.48, 0.89) | 0.007 |
| Cancer of brain and nervous system [35.] | 3.91 (1.40, 10.98) | 0.009 | Cancer of head and neck [11.] | 3.93 (2.53, 6.10) | <0.0001 |
| Cancer of ovary [27.] | 0.06 (0.01, 0.26) | <0.001 | Cancer of thyroid [36.] | 2.73 (1.27, 5.86) | 0.01 |
| Cancer of thyroid [36.] | 3.27 (1.20, 8.88) | 0.02 | Cardiac dysrhythmias [106.] | 1.15 (1.02, 1.29) | 0.017 |
| Cataract [86.] | 1.41 (1.19, 1.68) | <0.001 | Cataract [86.] | 1.40 (1.24, 1.59) | <0.0001 |
| Cerebrovascular anomalies | 9.45 (2.47, 36.07) | 0.001 | Cellulitis and abscess | 1.26 (1.06, 1.49) | 0.007 |
| Chronic airway obstruction; not otherwise specified | 0.63 (0.5, 0.8) | <0.001 | Cerebrovascular anomalies | 10.37 (3.7, 29.08) | <0.0001 |
| Chronic kidney disease [158.] | 0.70 (0.56, 0.89) | 0.004 | Chronic airway obstruction; not otherwise specified | 0.69 (0.60, 0.80) | <0.0001 |
| Codes related to mental health disorders [6631] | 1.65 (1.29, 2.12) | <0.0001 | Chronic kidney disease [158.] | 0.71 (0.60, 0.83) | <0.0001 |
| Codes related to substance-related disorders [6632] | 1.47 (1.12, 1.92) | 0.005 | Codes related to mental health disorders [6631] | 1.37 (1.14, 1.65) | <0.001 |
| Coma; stupor; and brain damage [85.] | 1.94 (1.43, 2.63) | <0.0001 | Codes related to substance-related disorders [6632] | 1.62 (1.37, 1.92) | <0.0001 |
| Concussion | 3.49 (2.05, 5.94) | <0.0001 | Coma; stupor; and brain damage [85.] | 1.39 (1.11, 1.74) | 0.004 |
| Conditions associated with dizziness or vertigo [93.] | 3.61 (3.03, 4.30) | <0.0001 | Complication of device; implant or graft [237.] | 0.71 (0.58, 0.88) | 0.001 |
| Conduct disorder [6521] | 0.13 (0.03, 0.56) | 0.006 | Conditions associated with dizziness or vertigo [93.] | 1.26 (1.10, 1.45) | 0.001 |
| Congenital anomalies of the eye | 7.78 (1.97, 30.75) | 0.003 | Constipation | 0.74 (0.61, 0.89) | 0.001 |
| Constipation | 0.73 (0.55, 0.97) | 0.03 | Crushing injury or internal injury [234.] | 5.27 (3.87, 7.17) | <0.0001 |
| Convulsions | 0.50 (0.33, 0.77) | 0.001 | Delirium dementia and amnestic and other cognitive disorders [653] | 0.72 (0.59, 0.87) | <0.001 |
| Coronary atherosclerosis and other heart disease [101.] | 0.74 (0.62, 0.88) | <0.001 | Depressive disorders [6572] | 0.79 (0.68, 0.92) | 0.002 |
| Crushing injury or internal injury [234.] | 9.89 (6.57, 14.87) | <0.0001 | Diabetes with neurological manifestations | 0.69 (0.55, 0.86) | <0.001 |
| Deficiency and other anemia [59.] | 0.82 (0.68, 0.99) | 0.035 | Disorders of lipid metabolism [53.] | 1.22 (1.09, 1.36) | <0.001 |
| Delirium dementia and amnestic and other cognitive disorders [653] | 0.52 (0.40, 0.68) | <0.0001 | Disorders of teeth and jaw [136.] | 1.44 (1.05, 1.97) | 0.023 |
| Depressive disorders [6572] | 0.72 (0.58, 0.88) | 0.002 | Dysphagia | 1.38 (1.13, 1.69) | 0.002 |
| Diverticulosis and diverticulitis [146.] | 1.41 (1.08, 1.84) | 0.012 | Elimination disorders [6551] | 44.06 (1.20, 1619.84) | 0.04 |
| Epilepsy | 0.61 (0.38, 0.97) | 0.035 | Epilepsy | 0.64 (0.48, 0.86) | 0.003 |
| Female infertility [174.] | 6.75 (0.70, 65.33) | 0.099 | Essential hypertension [98.] | 1.36 (1.20, 1.54) | <0.0001 |
| Gangrene [248.] | 4.55 (2.07, 10.01) | <0.001 | Fever of unknown origin [246.] | 0.68 (0.54, 0.86) | 0.001 |
| Genitourinary symptoms and ill-defined conditions [163.] | 0.77 (0.64, 0.93) | 0.005 | Heart valve disorders [96.] | 1.35 (1.19, 1.53) | <0.0001 |
| Hemiplegia | 2.92 (1.95, 4.36) | <0.0001 | Hemiplegia | 7.90 (6.03, 10.34) | <0.0001 |
| Hemorrhage of gastrointestinal tract | 0.54 (0.35, 0.84) | 0.006 | Hemorrhage of gastrointestinal tract | 0.62 (0.45, 0.86) | 0.004 |
| Hypopotassemia | 2.58 (1.93, 3.44) | <0.0001 | Hemorrhoids [120.] | 1.38 (1.09, 1.74) | 0.008 |
| Immunity disorders [57.] | 4.63 (3.95, 5.43) | <0.0001 | Hodgkins disease [37.] | 0.05 (0.01, 0.47) | 0.009 |
| Joint disorders and dislocations; trauma-related [225.] | 0.2 (0.14, 0.28) | <0.0001 | Hyposmolality | 12.8 (1.05, 155.54) | 0.045 |
| Menstrual disorders [171.] | 0.26 (0.09, 0.73) | 0.01 | Immunity disorders [57.] | 4.76 (4.27, 5.3) | <0.0001 |
| Multiple sclerosis [80.] | 1.76 (1.42, 2.17) | <0.0001 | Joint disorders and dislocations; trauma-related [225.] | 0.22 (0.17, 0.29) | <0.0001 |
| Neoplasms of unspecified nature or uncertain behavior [44.] | 0.46 (0.25, 0.83) | 0.01 | Lymphadenitis [247.] | 1.30 (1.16, 1.46) | <0.0001 |
| Nervous system congenital anomalies [216.] | 0.58 (0.37, 0.90) | 0.015 | Malposition; malpresentation [187.] | 1.20 (1.07, 1.34) | 0.002 |
| Non-Hodgkins lymphoma [38.] | 1.61 (1.16, 2.23) | 0.004 | Microtia (incomplete development of external ear) | 1.81 (1.39, 2.36) | <0.0001 |
| Normal delivery | 0.42 (0.28, 0.63) | <0.0001 | Non-Hodgkins lymphoma [38.] | 1.59 (1.27, 2.00) | <0.0001 |
| Obstructive chronic bronchitis | 4.02 (3.31, 4.88) | <0.0001 | Noninfectious gastroenteritis [154.] | 1.51 (1.34, 1.70) | <0.0001 |
| Other acquired deformities [209.] | 2.04 (1.61, 2.59) | <0.0001 | Nonspecific chest pain [102.] | 0.72 (0.6, 0.85) | <0.001 |
| Other and unspecified complications of birth; puerperium affecting management of mother | 1.28 (1.09, 1.50) | 0.003 | Obstructive chronic bronchitis | 3.74 (3.29, 4.25) | <0.0001 |
| Other and unspecified perinatal conditions | 1.76 (1.50, 2.07) | <0.0001 | Osteoarthritis [203.] | 1.21 (1.02, 1.42) | 0.025 |
| Other biliary tract disease | 1.52 (1.19, 1.96) | 0.001 | Osteogenesis imperfecta | 0.21 (0.06, 0.73) | 0.014 |
| Other congenital anomalies of genitalia | 0.04 (0.01, 0.26) | <0.001 | Other acquired deformities [209.] | 2.15 (1.80, 2.58) | <0.0001 |
| Other congenital anomalies of limbs | 7.88 (2.43, 25.54) | <0.001 | Other and ill-defined heart disease [104.] | 1.19 (1.04, 1.36) | 0.011 |
| Other congenital anomalies of the heart | 1.33 (1.13, 1.56) | <0.001 | Other and unspecified perinatal conditions | 1.17 (1.04, 1.31) | 0.008 |
| Other connective tissue disease [211.] | 2.40 (1.22, 4.71) | 0.011 | Other bacterial infections [3.] | 1.21 (1.06, 1.39) | 0.006 |
| Other female genital disorders [175.] | 14.55 (11.25, 18.83) | <0.0001 | Other biliary tract disease | 1.68 (1.4, 2.01) | <0.0001 |
| Other fractures [231.] | 3.74 (3.11, 4.51) | <0.0001 | Other chronic skin ulcer | 1.55 (1.38, 1.73) | <0.0001 |
| Other hereditary and degenerative nervous system conditions [81.] | 1.39 (1.05, 1.82) | 0.02 | Other congenital anomalies of the heart | 1.18 (1.06, 1.32) | 0.003 |
| Other infections; including parasitic [8.] | 1.61 (1.33, 1.96) | <0.0001 | Other endocrine disorders [51.] | 1.43 (1.26, 1.63) | <0.0001 |
| Other mycoses | 0.07 (0.02, 0.25) | <0.0001 | Other face and neck congenital anomalies | 1.29 (1.03, 1.61) | 0.026 |
| Other nervous system congenital anomalies | 1.98 (1.66, 2.34) | <0.0001 | Other female genital disorders [175.] | 3.07 (2.43, 3.86) | <0.0001 |
| Other nervous system disorders [95.] | 1.37 (1.06, 1.78) | 0.017 | Other fractures [231.] | 2.18 (1.89, 2.52) | <0.0001 |
| Other perinatal conditions [224.] | 2.83 (1.88, 4.25) | <0.0001 | Other malnutrition | 0.84 (0.73, 0.98) | 0.022 |
| Other respiratory congenital anomalies | 1.63 (1.38, 1.93) | <0.0001 | Other miscellaneous mental conditions [6709] | 4.44 (1.14, 17.33) | 0.032 |
| Other skin disorders [200.] | 0 (0, 7.57) | 0.134 | Other nervous system congenital anomalies | 1.44 (1.27, 1.63) | <0.0001 |
| Outcome of delivery (V codes) | 0.55 (0.35, 0.88) | 0.011 | Other nervous system disorders [95.] | 1.25 (1.05, 1.49) | 0.012 |
| Ovarian cyst [172.] | 0.53 (0.31, 0.90) | 0.018 | Other non-traumatic joint disorders [204.] | 0.50 (0.28, 0.90) | 0.02 |
| Parkinsons disease [79.] | 0.48 (0.30, 0.77) | 0.002 | Other obstetrical trauma | 1.80 (1.21, 2.68) | 0.004 |
| Patent ductus arteriosus | 25.85 (1.45, 462.14) | 0.027 | Other respiratory congenital anomalies | 1.38 (1.23, 1.55) | <0.0001 |
| Pectus excavatum | 0.68 (0.55, 0.84) | <0.001 | Other spinal congenital anomalies | 1.20 (1.07, 1.35) | 0.003 |
| Rectal and large intestine atresia/stenosis | 0.58 (0.45, 0.76) | <0.0001 | Other upper gastrointestinal congenital anomalies | 1.38 (1.22, 1.56) | <0.0001 |
| Regional enteritis and ulcerative colitis [144.] | 2.13 (1.79, 2.53) | <0.0001 | Parkinsons disease [79.] | 0.49 (0.32, 0.74) | <0.001 |
| Respiratory conditions of fetus and newborn; other than respiratory distress | 2.18 (1.53, 3.11) | <0.0001 | Persistent fetal circulation | 3.36 (1.22, 9.24) | 0.019 |
| Schizophrenia and other psychotic disorders [659] | 0.02 (0, 0.11) | <0.0001 | Pneumonia (except that caused by TB or STD) [122.] | 0.71 (0.53, 0.95) | 0.023 |
| Short gestation; low birth weight; and fetal growth retardation [219.] | 2.15 (1.22, 3.79) | 0.008 | Poisoning by other medications and drugs [242.] | 5.30 (1.57, 17.9) | 0.007 |
| Somatoform disorders [6707] | 26.79 (15.49, 46.33) | <0.0001 | Regional enteritis and ulcerative colitis [144.] | 1.50 (1.35, 1.68) | <0.0001 |
| Spondylosis and allied disorders | 1.26 (1.00, 1.59) | 0.048 | Respiratory conditions of fetus and newborn; other than respiratory distress | 2.24 (1.77, 2.84) | <0.0001 |
| Sprains and strains [232.] | 0.39 (0.22, 0.67) | <0.001 | Respiratory failure | 1.17 (1.02, 1.35) | 0.029 |
| Thyrotoxicosis with or without goiter | 1.88 (1.47, 2.41) | <0.0001 | Schizophrenia and other psychotic disorders [659] | 0.11 (0.04, 0.35) | <0.001 |
|  |  |  | Sexual and gender identity disorders [6705] | 2.09 (1.37, 3.19) | <0.001 |
|  |  |  | Short gestation; low birth weight; and fetal growth retardation [219.] | 5.25 (3.35, 8.24) | <0.0001 |
|  |  |  | Suicide and intentional self-inflicted injury [662] | 1.55 (1.31, 1.84) | <0.0001 |
|  |  |  | Thyrotoxicosis with or without goiter | 1.60 (1.34, 1.91) | <0.0001 |

| **Table S2. Ischemic Stroke Controls Odds Ratios for Variables used in the Multi-variable Adjustment** | | | | | | | |
| --- | --- | --- | --- | --- | --- | --- | --- |
| **Vertebral Artery Dissection** | | | **Carotid Artery Dissection** | | |  |  |
|  | **Odds Ratio (95% CI)** | **p. Value** |  | **Odds Ratio (95% CI)** | **p. Value** |  |  |
| **Age** | 0.97 (0.97, 0.98) | 5.47E-83 | **Age** | 0.97 (0.97, 0.97) | 1.29E-208 |  |  |
| **Sex** |  |  | **Sex** |  |  |  |  |
| Male | Ref |  | Male | Ref |  |  |  |
| Female | 0.74 (0.69, 0.79) | 7.59E-21 | Female | 0.85 (0.81, 0.89) | 4.17E-11 |  |  |
| **Race** |  |  | **Race** |  |  |  |  |
| White | Ref |  | White | Ref |  |  |  |
| Black | 0.57 (0.51, 0.63) | 4.38E-25 | Black | 0.78 (0.72, 0.84) | 2.84E-11 |  |  |
| Asian | 1.32 (1.08, 1.60) | 0.0054 | Asian | 1.20 (1.00, 1.41) | 0.042 |  |  |
| Hispanic | 0.87 (0.70, 1.07) | 0.215 | Hispanic | 0.86 (0.72, 1.02) | 0.101 |  |  |
| North American Native | 1.01 (0.68, 1.44) | 0.947 | North American Native | 0.67 (0.44, 0.96) | 0.0406 |  |  |
| Other | 1.46 (1.18, 1.77) | 0.000231 | Other | 1.22 (1.01, 1.46) | 0.0309 |  |  |
| **CCS Diagnosis Groups** |  |  | **CCS Diagnosis Groups** |  |  |  |  |
| Hemorrhage during pregnancy; abruptio placenta; placenta previa [182.] | 3.59 (1.08, 8.76) | 0.0138 | Digestive congenital anomalies [214.] | 20.62 (0.69, 244.78) | 0.0406 |  |  |
| Spinal cord injury [227.] | 3.36 (2.63, 4.22) | 8.88E-24 | Normal delivery | 4.65 (1.39, 11.60) | 0.0036 |  |  |
| Late effects of cerebrovascular disease [113.] | 0.25 (0.20, 0.32) | 8.02E-31 | Aortic; peripheral; and visceral artery aneurysms [115.] | 3.37 (3.14, 3.61) | 1.75E-255 |  |  |
| Acute cerebrovascular disease [109.] | 0.35 (0.30, 0.41) | 9.68E-36 | All other congenital anomalies | 2.42 (1.96, 2.95) | 4.14E-17 |  |  |
| Attention deficit disorder and Attention deficit hyperactivity disorder [6523] | 1.71 (1.16, 2.40) | 0.00383 | Acute cerebrovascular disease [109.] | 0.38 (0.34, 0.43) | 1.64E-58 |  |  |
| Other contraceptive and procreation management | 1.88 (1.11, 2.97) | 0.0115 | Delirium dementia and amnestic and other cognitive disorders [653] | 0.62 (0.56, 0.67) | 6.24E-26 |  |  |
| Aortic; peripheral; and visceral artery aneurysms [115.] | 1.98 (1.77, 2.21) | 1.82E-33 | Late effects of cerebrovascular disease [113.] | 0.40 (0.34, 0.46) | 1.04E-35 |  |  |
| Delirium dementia and amnestic and other cognitive disorders [653] | 0.71 (0.63, 0.78) | 1.44E-10 | Migraine | 1.28 (1.13, 1.45) | 8.30E-05 |  |  |
| Transient cerebral ischemia [112.] | 0.58 (0.49, 0.67) | 1.55E-11 | Cancer of head and neck [11.] | 1.99 (1.67, 2.35) | 1.79E-15 |  |  |
| Congestive heart failure; nonhypertensive [108.] | 0.82 (0.74, 0.90) | 2.59E-05 | Other contraceptive and procreation management | 1.50 (0.98, 2.21) | 0.0487 |  |  |
| Occlusion or stenosis of precerebral arteries [110.] | 0.66 (0.59, 0.73) | 2.17E-13 | Congestive heart failure; nonhypertensive [108.] | 0.82 (0.77, 0.87) | 2.19E-09 |  |  |
| Sprains and strains [232.] | 1.26 (1.15, 1.37) | 2.36E-07 | Other screening for suspected conditions (not mental disorders or infectious disease | 1.23 (1.17, 1.30) | 8.91E-17 |  |  |
| Other male genital disorders [166.] | 1.17 (1.04, 1.32) | 0.0109 | Medical examination/evaluation [256.] | 1.21 (1.15, 1.27) | 7.36E-13 |  |  |
| Deficiency and other anemia [59.] | 0.81 (0.76, 0.88) | 3.73E-08 | Other mycoses | 0.92 (0.87, 0.98) | 0.0139 |  |  |
| Other back problems | 1.19 (1.11, 1.28) | 1.66E-06 | Urinary tract infections [159.] | 0.89 (0.84, 0.95) | 0.000273 |  |  |
| Urinary tract infections [159.] | 0.83 (0.77, 0.90) | 2.45E-06 | Menstrual disorders [171.] | 1.28 (1.00, 1.62) | 0.0451 |  |  |
| Other headache | 1.28 (1.17, 1.40) | 2.01E-08 | Other and unspecified benign neoplasm [47.] | 1.20 (1.13, 1.27) | 1.12E-09 |  |  |
| Medical examination/evaluation [256.] | 1.19 (1.12, 1.27) | 6.28E-08 | Codes related to substance-related disorders [6632] | 0.94 (0.87, 1.01) | 0.112 |  |  |
| Bipolar disorders [6571] | 1.09 (0.93, 1.28) | 0.282 | Other male genital disorders [166.] | 1.22 (1.11, 1.35) | 7.30E-05 |  |  |
| Other mycoses | 0.82 (0.76, 0.90) | 6.24E-06 | Other headache | 1.19 (1.11, 1.28) | 7.30E-07 |  |  |
| Chronic airway obstruction; not otherwise specified | 0.76 (0.69, 0.83) | 3.67E-09 | Diabetes mellitus without complication [49.] | 0.81 (0.77, 0.85) | 1.20E-16 |  |  |
| Spondylosis and allied disorders | 1.22 (1.11, 1.34) | 1.75E-05 | Other upper respiratory infections [126.] | 1.14 (1.08, 1.21) | 8.87E-06 |  |  |
| Cardiac dysrhythmias [106.] | 0.86 (0.81, 0.93) | 2.74E-05 | Chronic kidney disease [158.] | 0.83 (0.77, 0.89) | 3.94E-07 |  |  |
| Codes related to mental health disorders [6631] | 1.33 (1.19, 1.47) | 2.73E-07 | Deficiency and other anemia [59.] | 0.92 (0.87, 0.97) | 0.00408 |  |  |
| Hypertension with complications and secondary hypertension [99.] | 0.87 (0.79, 0.95) | 0.00216 | Essential hypertension [98.] | 1.00 (0.95, 1.06) | 0.975 |  |  |
| Other screening for suspected conditions (not mental disorders or infectious disease | 1.14 (1.07, 1.21) | 2.38E-05 | Attention deficit disorder and Attention deficit hyperactivity disorder [6523] | 1.58 (1.12, 2.15) | 0.0058 |  |  |
| Coronary atherosclerosis and other heart disease [101.] | 0.85 (0.80, 0.91) | 5.35E-06 |  |  |  |  |  |
| Peripheral and visceral atherosclerosis [114.] | 0.90 (0.83, 0.98) | 0.0116 |  |  |  |  |  |
| Malaise and fatigue [252.] | 0.84 (0.79, 0.90) | 8.55E-07 |  |  |  |  |  |
| Hyperplasia of prostate [164.] | 1.09 (0.99, 1.19) | 0.0722 |  |  |  |  |  |
| Cancer of bronchus; lung [19.] | 0.51 (0.38, 0.68) | 8.12E-06 |  |  |  |  |  |
| Essential hypertension [98.] | 0.99 (0.93, 1.06) | 0.786 |  |  |  |  |  |
| Diabetes mellitus without complication [49.] | 0.83 (0.78, 0.88) | 1.42E-09 |  |  |  |  |  |
| Migraine | 0.98 (0.83, 1.16) | 0.835 |  |  |  |  |  |
| Intervertebral disc disorders | 1.03 (0.94, 1.12) | 0.569 |  |  |  |  |  |
| Other and unspecified benign neoplasm [47.] | 1.06 (0.99, 1.15) | 0.108 |  |  |  |  |  |
| Immunizations and screening for infectious disease [10.] | 1.21 (1.14, 1.28) | 1.99E-10 |  |  |  |  |  |
| Personality disorders [658] | 1.31 (0.93, 1.79) | 0.103 |  |  |  |  |  |
| Blindness and vision defects [89.] | 1.18 (1.09, 1.28) | 3.75E-05 |  |  |  |  |  |
| Other bone disease and musculoskeletal deformities [212.] | 1.09 (1.01, 1.18) | 0.022 |  |  |  |  |  |
| Other malnutrition | 1.28 (1.18, 1.38) | 1.82E-09 |  |  |  |  |  |
| Other skin disorders [200.] | 1.18 (1.10, 1.25) | 4.39E-07 |  |  |  |  |  |
| Obstructive chronic bronchitis | 0.76 (0.65, 0.89) | 0.000503 |  |  |  |  |  |
| Other upper respiratory infections [126.] | 1.07 (1.00, 1.15) | 0.0501 |  |  |  |  |  |
| Melanomas of skin [22.] | 1.35 (1.09, 1.66) | 0.00526 |  |  |  |  |  |
| Anxiety disorders [651] | 1.08 (0.99, 1.18) | 0.0819 |  |  |  |  |  |
| Chronic kidney disease [158.] | 0.88 (0.79, 0.97) | 0.0114 |  |  |  |  |  |
| *CCS covariates were identified through LASSO regression.* | | |  |  |  |  |  |
